# Controlling an Unprecedented Outbreak with Vancomycin-Resistant *Enterococcus faecium* in Germany, October 2015 to November 2019

**DOI:** 10.3390/microorganisms10081603

**Published:** 2022-08-09

**Authors:** Jennifer K. Bender, Julia Hermes, Lutz T. Zabel, Sebastian Haller, Nadja Mürter, Hans-Peter Blank, Guido Werner, Ingo Hüttner, Tim Eckmanns

**Affiliations:** 1 Nosocomial Pathogens and Antibiotic Resistances Unit, Department of Infectious Diseases, Robert Koch Institute, 38855 Wernigerode, Germany; 2 Healthcare-Associated Infections, Surveillance of Antibiotic Resistance and Consumption Unit, Department of Infectious Disease Epidemiology, Robert Koch Institute, 13353 Berlin, Germany; 3 Alb Fils Kliniken GmbH, 73035 Göppingen, Germany; 4 Local Health Authority Göppingen, 73033 Göppingen, Germany; 5 Charité Comprehensive Cancer Center, 10117 Berlin, Germany

**Keywords:** vancomycin-resistant enterococci, hospital outbreak, transmission, whole-genome analysis, infection prevention and control measures

## Abstract

Hospital outbreaks with vancomycin-resistant enterococci (VRE) pose a serious health threat and a challenge to infection prevention and control (IPC). We herein report on a VRE outbreak of unprecedented extent in Southern Germany (October 2015–November 2019). We used descriptive epidemiology and whole-genome sequencing (WGS) for a detailed outbreak investigation. Of the 2905 cases, 2776 (95.3%) were colonized, whereas from 127 (3.7%), VRE could be isolated from otherwise sterile body fluids or sites unlikely for enterococci colonization. Cases had a median age of 78 years (IQR 68–84) and 1339/2905 (46%) were female. The majority of isolates sequenced belonged to the clonal lineage ST80/CT1013 (212/397, 53%). Nosocomial transmission was observed as well as the constant import of VRE into the hospital. Extensive IPC measures were implemented and terminated the outbreak in late 2019, eventually. Our study shows that the combination of epidemiological and genomic analyses is indispensable for comprehensive outbreak investigations. The adaptation of IPC measures to these findings, their timely implementation, and strict execution also allow containment of large VRE outbreaks in hospital settings.

## 1. Introduction

Enterococci are part of the intestinal flora of humans and animals alike and are usually considered harmless commensals. However, upon disruption of gastrointestinal homeostasis, colonization can progress to invasive infection, potentially eliciting bacterial endocarditis, bloodstream infections, urinary tract infections, wound infections, peritonitis, or intra-abdominal abscesses [1]. Enterococci are considered a leading cause of hospital-acquired infections [2], with the species *E. faecalis* and *E. faecium* being of major importance. The latter species often acquires glycopeptide (vancomycin) resistance, hence leaving limited treatment options in the case of severe infections. As a consequence, the WHO has listed vancomycin-resistant enterococci (VRE, mainly *E. faecium*) as high-priority pathogens for which new antibiotics are urgently needed [3].

The prevalence of VRE in European countries varies greatly, ranging from less than 1% VRE amongst invasive isolates in France or the Netherlands up to 57% in Lithuania in 2020 (https://atlas.ecdc.europa.eu/public/index.aspx?Dataset=27&HealthTopic=4, accessed on 30 June 2022). Especially within Germany, VRE are on the rise and their prevalence has more than doubled from 2015 to 2020 (10.5% to 22%) (https://atlas.ecdc.europa.eu/public/index.aspx, accessed on 30 June 2022). Outbreaks and clusters with VRE have been widely reported [4,5,6,7,8]. A systematic review on VRE outbreaks in hematology and oncology departments assessed 35 outbreaks involving 10–19 patients, with one study having 124 patients affected at maximum [9]. In Germany, the number of notified outbreaks with *Enterococcus* spp. increased from 5 in 2015 to 24 in 2021 with a maximum of 49 cases per outbreak (2021) [Robert Koch Institute (RKI), personal communications]. This represents a worrisome trend given that only nosocomial infections occurring in ≥2 individuals with a putative epidemiological connection are notifiable according to the German Protection against Infection Act and, thus, total numbers are strongly underestimated.

A country-wide awareness for VRE challenging German hospitals was also noted by the National Reference Centre (NRC) for Staphylococci and Enterococci at the RKI, demonstrating that the majority of nosocomial outbreaks are caused by specific *E. faecium* lineages that belong to the clade of hospital-associated enterococci [10,11]. Those lineages must be discerned from commensal or animal-associated isolates and likely adapted to hospital settings [12], although a genetic continuum exists between these clades.

For efficient containment of VRE, it is well known that the success of single Infection Prevention and Control (IPC) measures is difficult to dissect and a bundle of measures such as screening, intensified environmental cleaning, and hygiene education are usually implemented to control VRE influx and/or transmission in hospital environments [8,13]. In 2018, the German Commission for Hospital Hygiene and Infection Prevention (KRINKO) formulated a corresponding recommendation for hygiene measures for the prevention of infection by enterococci with specific antibiotic resistances [14].

Here, we report on a joined effort of epidemiologists, microbiologists, hygiene specialist, clinicians, and local health officers to control an unprecedented outbreak of VRE affecting two regional hospitals in Southern Germany from 2015 until 2019. We aim to comprehensively describe the outbreak by means of epidemiological and genomic data, and to summarize IPC measures that resulted in termination of the outbreak, eventually.

## 2. Materials and Methods

### 2.1. Setting

The two hospitals affected (hospital X and Y) belong to one trust and represent the largest healthcare institution in that region. The hospitals comprise 20 specialized units and all levels of care. They provide a total of 775 beds with about 3300 inpatients and 120,000 outpatients per year. An in-house department for laboratory medicine is responsible for diagnostics.

### 2.2. Definitions

We defined a VRE case as a patient who presented at either hospital and from whom a VRE, either colonization or clinical specimen, was extracted between 1 October 2015 (rapid increase in case numbers) and 19 November 2019 (official end date of the outbreak).

We defined VRE clinical specimens as VRE obtained from material that is supposed to be of sterile nature or unlikely to be the site of *E. faecium* colonization such as blood, surgical sites, or urine. VRE from all other materials, e.g., from rectal swabs and stool cultures, were defined as VRE colonization.

Nosocomial acquisition or transmission was defined as VRE (colonization or infection) detection >48 h after admission of the case-patient to either hospital.

### 2.3. Epidemiological Analyses

Descriptive epidemiology was carried out using patient information on age, sex, date of admission and discharge, death (due to or not due to VRE), previous admission during the outbreak period to either one of the hospitals, material and date of material extraction, and date of VRE reporting.

We assessed the timeliness of reporting for cases with values >0 and <64 days between the date of material extraction and date of reporting, as values beyond were considered implausible. For determining nosocomial VRE acquisition, only those cases that tested positive >0 and <150 days between hospital admission and VRE extraction were considered. The same holds true for calculating the average length of stay, as a hospital stay of more than 150 days was highly unlikely and possibly due to documentation errors.

Analyses were carried out in RStudio 2022.2.3.492.

### 2.4. Infection Prevention and Control (IPC) Measures

Up until 2015, screening by anal swabs was performed according to the hospital’s routine hygiene plan on intensive care units only or for the purpose of surveillance of VRE-positive patients. The screening regime was later adapted as outlined in Section 3.4.1. In addition, a point prevalence screening was conducted from 22 to 24 May 2017 in hospital X, and from 29 to 31 May 2017 in hospital Y. Multiple IPC measures were implemented stepwise as outlined in Supplementary Table S1. Briefly, these comprised providing detailed information about VRE to staff, patients, and visitors; disinfection; cleaning; hand hygiene, reconstruction measures and patient isolation.

### 2.5. Laboratory Methods

Detection and isolation of VRE were performed at the hospital’s department for laboratory medicine. VRE were isolated by streaking patient specimens on VRE-selective media (Mast Diagnostica GmbH, Reinfeld, Germany) and inoculating Schaedler broth (BD, Heidelberg, Germany) as enrichment broth. Cultures were incubated at 36 ± 2 °C at least for 2 days. In case of the absence of growth of enterococci on solid media, 10 µL of the enrichment broth was plated on VRE-selective media and incubated as mentioned above. Suspect colonies were identified by MALDI TOF (Vitek MS, BioMerieux, Nuertingen) and resistance patterns were assessed by using Vitek (BioMerieux, Nuertingen, Germany), E-test (BioMerieux, Nuertingen, Germany), or agar diffusion testing, all according to CLSI testing standards. Indeterminate results were checked by using molecular detection of the *vanA* and *vanB* gene (BioGX/Axonlab, Ebersbach, Germany). Every first VRE-strain from the affected patients and every strain, obtained from expectantly sterile body sites, was conserved at −80°C for further research.

Typing of isolates from 391/2905 (13%) case-patients was performed at the NRC for Staphylococci and Enterococci at RKI. Due to limited sequencing capacities, we defined the following categories of isolates that were selected for whole-genome sequencing (WGS): C1, the first isolates obtained since 1 October 2015 until 15 January 2016 of which a sample was retained (*n* = 44); C2, all isolates of cases who either had a negative test on admission and were VRE-positive at discharge OR who had their first VRE sample collected at the date of discharge (with no information on VRE status at admission) AND who have not had any previous stay or contact with either hospital during the outbreak period (collectively summarized as C2; *n* = 82); C3, all isolates of cases from whom the first VRE sample was collected on the date of admission AND who have not had any previous stay or contact with either hospital since the beginning of the outbreak (*n* = 55). C1-C3 isolates were obtained between 2015 and 2017. In addition, C4 isolates were collected between December 2018 and March 2019; they correspond per definition to C2/C3 isolates but were collected irrespective of a previous stay of the patient at one of the hospitals (*n* = 53 and *n* = 37). We additionally typed all isolates from patients newly identified by the point prevalence study (PPS) in 2017 (*n* = 35). Moreover, from 11 patients, we sequenced two isolates, later named “isolate pairs”, one obtained from rectal swabs and one obtained from supposedly sterile body fluids (*n* = 22). We also sequenced isolates from clinical specimens (isolates found in expectantly noncolonized samples), some of which were also assigned to C1, C2, C3, or isolate pairs, respectively (*n* = 111 in total, but only 73 were not assigned to another category). One additional isolate was obtained in June 2018 (GP435) and did not fall in any of the above-mentioned categories but was kept for further analysis of clinical specimens vs. colonization isolates.

To obtain WGS data, bacteria were grown overnight in Mueller–Hinton broth and DNA was isolated according to the manufacturer’s instructions (DNeasy Blood and Tissue Kit, Qiagen, Hilden, Germany). Bacterial DNA was subjected to library preparation using the Nextera XT library prep kit (Illumina^®^, San Diego, CA, USA) and sequenced at the sequencing facility MF2 of the RKI on MiSeq and HiSeq platforms with a read out of 2 × 300 bp and 2 × 250 bp, respectively. Genome reconstruction was achieved by utilizing the in-house-generated bioinformatics pipelines QCumber (for quality check; https://gitlab.com/RKIBioinformaticsPipelines/QCumber, accessed on 30 June 2022) and batchAssembly.py (for whole-genome reconstruction). The reconstructed sequences were further analyzed using the SeqSphere+^®^ software suite (Ridom GmbH, Münster, Germany). Sequence types (STs) and complex types (CTs) were inferred, the latter of which is based on a core genome multi-locus sequencing typing (cgMLST) scheme comprising 1423 core genes and implemented in the SeqSphere+ software. The description of genetic relatedness was carried out by calculating minimum spanning trees (MSTs) and neighbor joining trees (NJTs). The NJT is based on Nei’s *D_A_* distance [15] comparing alleles of isolates. The resulting distance matrix is provided in Supplementary Table S2. Isolates were analyzed by pairwise comparison and assigned to a cluster of highly related strains if the difference was ≤15 alleles [16]. The vancomycin genotype was either verified by PCR or by inferring the vancomycin resistance locus from the NCBI AMR Finder utilizing WGS data.

### 2.6. Ethics

All case information was collected by the hospital. This outbreak investigation was conducted as part of an administrative assistance procedure under §16 of the German Protection against Infection Act and as requested by the state health authority and, thus, is exempt from ethical approval.

## 3. Results

### 3.1. Outbreak Description

In October 2015, an unusually high number (*n* = 13) of VRE isolated from patient specimens was noticed at the department of laboratory medicine of two interrelated hospitals (hospital X and Y) in Southern Germany. VRE isolation exceeded that of previous months when nine cases were detected between January and September 2015 (not shown).

**Figure 1:**
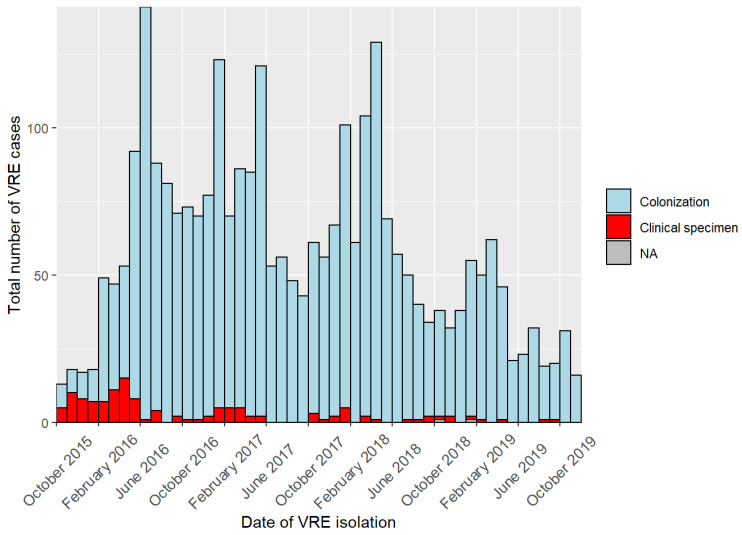
Number of cases in a nosocomial outbreak with VRE in two interconnected hospitals in Southern Germany, October 2015 to November 2019. Number of cases are displayed per month. Colors delineate where VRE isolates were obtained from, either colonization (=rectal swab; blue) or clinical specimens (=supposedly sterile body fluids, such as blood; red). NA = no information about source of isolation.

The outbreak was notified to the local public health authority (LPHA) in March 2016. In May 2016, the German Central Public Health Institute, RKI, received the first notification of a nosocomial cluster of VRE affecting the two hospitals X and Y. The LPHA subsequently initiated an outbreak investigation and requested strict IPC measures such as general screening upon admission, case-patient isolation, or implementation of an antibiotic stewardship (ABS) team. However, and presumably due to the fact that, in the beginning, IPC measures were not strictly followed to the full extent, cases were continuously reported for a duration of four years (Figure 1). The outbreak was officially declared over on 19 November 2019, when no VRE clinical specimens were detected for the duration of more than two months. In the period between 1 October 2015 and 19 November 2019, 2905 individuals were tested positive for VRE (Figure 1).

### 3.2. Epidemiological Investigation

Cases had a median age of 78 years (IQR 68–84) and 1339/2905 (46%) were female. Of 2903 cases, 2776 (96%) persons were colonized with enterococci, whereas from 127 (4%) patients, VRE could be isolated from otherwise sterile body fluids (Figure 1). The majority of isolates, 2733/2903 (94%), were obtained from rectal swabs. Sex was not associated with an increased risk for VRE from sterile body fluids (chi-squared test *p* = 0.22). Deceased status was available for 1568 cases of which 495 (31%) VRE case-patients died during their hospital stay; 239/495 (48%) were female. For 309/495 deceased VRE case-patients, information was available whether their death could be attributed to VRE; it was reported positive for 0.6% (2/309).

Assuming hospital acquisition if VRE were extracted >48 h upon admission, 1550/2784 (56%) of cases acquired VRE in the hospital. However, it must be noted that this includes cases that were tested after 48 h for the first time, hence overestimating the true number of hospital-acquired VRE. For a more detailed analysis, we had information about previous contact with either hospital for 2893 patients. Of those with a time span of less than 150 days between admission and VRE extraction (see Methods for explanation), 637/1876 (34%) with previous contact were positive on the day of admission. Most importantly, 579/896 (65%) with no contact with the hospital acquired VRE after 48 h, hence emphasizing the dramatic VRE situation within the two clinics. In addition, the percentage of VRE from clinical specimens was high for those with no previous admission and nosocomial acquisition (8%, 48/579).

Case-patients stayed at the hospital for a median of 10 days (IQR 6–19). Of all cases with information, 1974/2893 (68%) were previously admitted to either one of the hospitals since 1 October 2015.

### 3.3. Typing of VRE Isolates

We sequenced a total of 402 isolates from 391 case-patients. We excluded five isolates from our analysis (three from category C3 and two from C4): two yielded no sequences, two turned out to be non-*E. faecium* species (*E. gallinarum*), and for one isolate, no sequence type could be inferred. It must be noted that four isolates were determined vancomycin-susceptible enterococci (VSE) and did not carry either *vanA* or *vanB* according to PCR and/or WGS results (not shown). This became apparent only after having examined selected isolates, and because we cannot rule out that the resistance gene cluster was lost between isolation of the strain at the hospital and typing at the NRC, we did not exclude the four VSE from our initial epidemiological assessment.

#### 3.3.1. Population Dynamics

**Figure 2:**
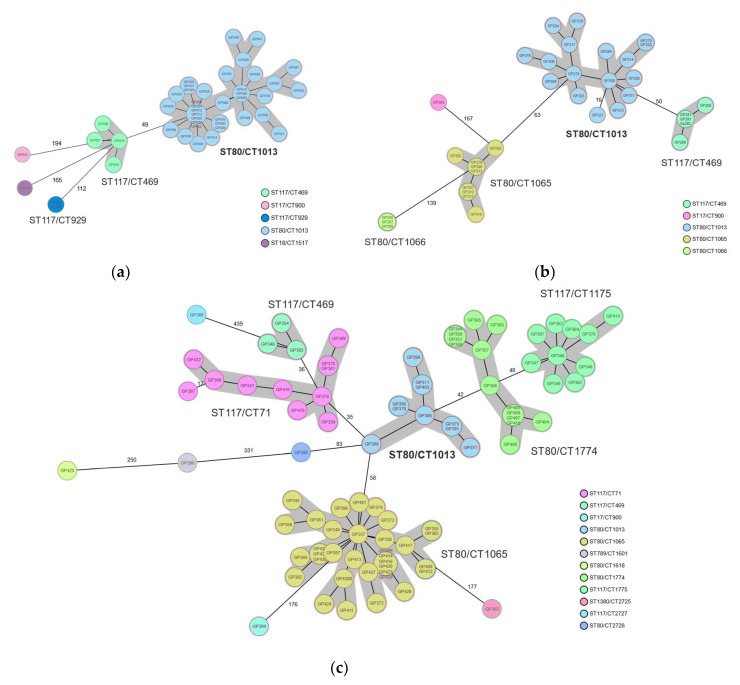
Genotyping results of VRE isolates from a nosocomial outbreak in two interconnected hospitals in Southern Germany, October 2015–November 2019. (**a**) Core genome multi-locus sequencing typing at the beginning of the outbreak 2015–2016. The large cluster of ST80/CT1013 is highlighted in blue. (**b**) Genotype distribution of isolates obtained from a point prevalence study in May 2017. (**c**) Genotypic assessment of isolates obtained between December 2018 and March 2019. Color-coding according to complex types (CT) based on the cgMLST nomenclature. Sequence types (ST) and CTs are shown adjacent to the clusters, highlighted in grey, which represent closely related isolates (difference ≤ 15 alleles). Numbers adjacent to connecting lines represent allelic differences between isolates with variation in more than 15 alleles.

In order to understand the clonality (or diversity) of the VRE population causing this large outbreak, we selected isolates according to five categories (C1–C4 and PPS, see Methods). The majority of the isolates harbored the *vanB* genotype 389/397 (98%) and four contained *vanA*. Multi-locus sequence typing (ST) and core genome multi-locus sequence typing (cgMLST, CT) of the first 44 isolates (=C1), obtained between 1 October 2015 and 15 January 2016, revealed the presence of a large cluster of ST80/CT1013 (36/44 (81%)) at the beginning of the outbreak (Figure 2a). A minor cluster of ST117/CT469 (*n* = 4) was also detected.

Isolates from patients newly identified by the point prevalence study (*n* = 35) in May 2017 revealed the presence of four different clusters containing >2 isolates (Figure 2b). Isolates with genotype ST80/CT1013 still represented the major outbreak clone (17/35 (49%)); however, additional clusters of ST80/CT1065 (9/35 (26%)), ST80/1066 (3/35 (9%)), and ST117/CT469 (5/35 (14%)) were detected (Figure 2b). In order to assess the population dynamics over time, we additionally sequenced 88 isolates obtained between December 2018 and March 2019 (=C4). As evident from Figure 2c, a shift in genotypes was observed. Although lineage ST80/CT1013, the prominent lineage at the very beginning of the outbreak and throughout 2017 (Figure 2a,b), was still detected, it no longer accounted for the majority of isolates. Now, genotype ST80/CT1065 (34/88 (39%)), which had previously represented a sub-cluster (Figure 2b), was the dominant clone recognized. Further, minor clusters of already known genotypes or entirely novel ones such as ST80/CT1774 and ST117/CT1775 were present (Figure 2b,c).

Isolates from clinical specimens were not restricted to a single clonal lineage but rather observed in all clusters detected over the years (Figure S1). It must be noted that we did not sequence all isolates from clinical specimens or colonization. Therefore, the ratio of clinical specimens to colonization isolates in certain clusters is most likely biased.

To evaluate intra-person VRE variability, we collected rectal swabs and clinical specimens of 11 patients. It is worth noting that 5/11 VRE pairs were collected 3–12 months apart; for one isolate pair, the second isolate (clinical specimen) was collected 34 months after the first. The majority of the two isolates from the same patient displayed the exact same ST/CT combination. However, 3/11 (27%) had different ST/CTs and showed 52, 55, and 379 allele differences (not shown). Of those, only two were collected with a three to four month timespan between VRE sampling from rectal swabs and clinical specimens. The isolate pair with 34 months between collection of the two samples showed 19 allele differences in corresponding cgMLST analysis (not shown).

#### 3.3.2. Nosocomial Transmission versus VRE Influx

**Figure 3:**
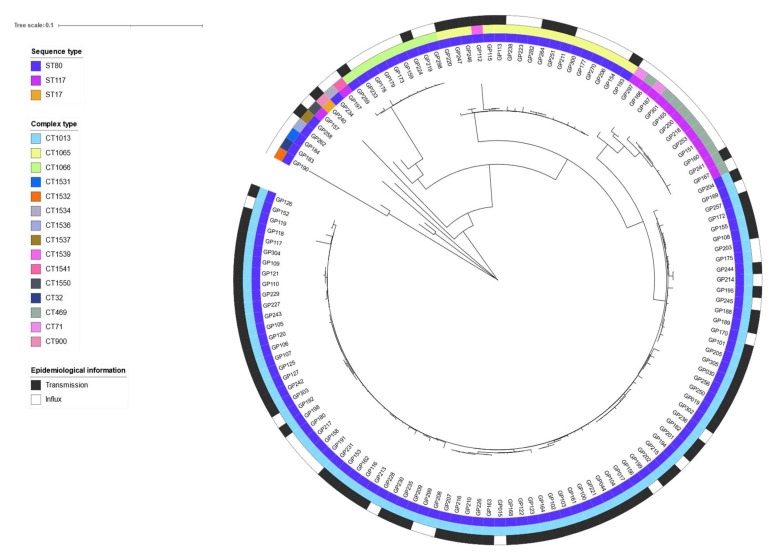
Differentiation between nosocomial transmission and VRE influx in an outbreak in two interconnected hospitals in Southern Germany, October 2015–November 2019. The neighbor joining tree based on Nei’s *D_A_* distance depicts the genetic relatedness of 134 isolates for which information on hospital-acquisition or VRE-positivity upon admission was available. The inner ring represents the sequence type (ST), the middle ring represents the complex type (CT), and the outer ring represents the epidemiological information on VRE transmission (black) and VRE influx (white). Tree visualization was realized by using the free online tool iTol v6 (https://itol.embl.de/, accessed on 30 June 2022).

We further wanted to understand whether the outbreak was due to VRE transmission within or the constant influx of strains/clones into the hospitals. Thus, we selected VRE isolates based on the date a patient was tested positive for VRE; that was either on admission (=VRE import) or upon discharge (=VRE transmission). WGS and genotyping were performed on 134 isolates obtained between 2016 and 2017 and for which such information was available. When combining epidemiological information with the genotyping results, it became obvious that the majority of isolates, 86/134 (64%), belonging to the main cluster of ST80/CT1013, were acquired after admission of the patients to either one of the hospitals and, hence, corresponds most likely to VRE transmission (Figure 3).

In contrast, minor clusters at the time of sampling, such as ST117/CT469, were mostly due to constant influx or re-import into the clinics as these patients tested VRE-positive on the day of admission (=VRE import) (Figure 3). It must be noted that 16/134 (12%) of these case-patients had been previously admitted to the hospitals during the outbreak period and were either negative upon admission or had not been tested.

### 3.4. Infection Prevention and Control Measures

#### 3.4.1. Screening Regime

Due to the continuous increase in case numbers (Figure 1), screening procedures were adjusted several times during the course of the outbreak and could be summarized as follows:

1. February 2016: screening of patients on wards of risk (nephrology, intensive care unit).
2. April 2016: extension of screening to patients on the hematology and gastroenterology ward.
3. May 2016: establishment of indicator wards with screening of all incoming and discharged patients (hematology, nephrology, visceral surgery, and gastroenterology).
4. May 2017: surveillance was intensified to screening patients upon admission and discharge on indicator wards and for any patient presenting with a previous stay at hospital X or Y during the outbreak period. Indicator wards included the hematology/oncology, palliative care, infectious diseases, nephrology, and visceral surgery units. Since then, weekly screening statistics were documented and are summarized in Supplementary Figures S2 and S3 for the period of May 2017 until the end of the outbreak in November 2019 (Figures S2 and S3).
5. July 2017: in consequence of the point prevalence study, intensified surveillance was expanded to all patients admitted to the hospitals regardless to which unit they were admitted or whether they had a previous stay in either hospital.
6. December 2017: in order to save resources, complete admission screening was replaced by a checklist-based screening of selected risk groups such as contact patients and patients that had been isolated or treated in either hospital before.
7. February 2018: due to low screening efficiency, full admission screening was re-implemented.
8. July 2019 until end of outbreak: checklist-based screening. Adherence was controlled by newly implemented IT-tools.

Although screening adherence on indicator wards was close to 100% (Figure S2), the VRE-positivity rate especially on indicator wards remained high and reached 13.5% in mid-April 2018 (Figure S3).

A high positivity rate was also obvious from the point prevalence study conducted in May 2017, when a total of 632/696 inpatients (91%) were screened at both hospitals. Of those, 71/632 (11%) were VRE-positive. In addition to the expected findings of known VRE cases, 35/71 (49%) were newly identified by the survey. Of those newly diagnosed cases, 23/35 (66%) previously had contact with either hospital after 1 October 2015.

We determined a median duration of 3 days (IQR 2–3) from VRE extraction until the day of the report. It is worth noting that patients awaiting their results were isolated to further prevent potential transmission.

#### 3.4.2. Other IPC Measures

A bundle of IPC measures was implemented to already existing rules throughout the course of the outbreak, some of which are named hereinafter and explained in more detail in Supplementary Table S1: Providing information about VRE, disinfection, cleaning, hand hygiene, reconstruction, and patient isolation.

## 4. Discussion

We herein conducted a detailed epidemiological and microbiological investigation of the largest VRE outbreak that has been reported up until today. The outbreak started in 2015, lasted four years, and comprised more than 2900 cases before it was officially declared over. In general, VRE outbreaks are no longer considered a rare event and have been reported from multiple countries in recent years [17,18,19,20,21,22,23], although not to this case-patient extent.

With respect to the vancomycin resistance genotype, *vanB* was the most frequently detected genetic locus in our study and present in all major outbreak clusters detected (98%). The population structure of *E. faecium* is quite variable and vancomycin resistance, either *vanA*- or *vanB*-type resistance, adds to the broad diversity of VRE and difficulty with respect to comparing outbreaks on a genetic basis. It has been demonstrated that hospital-associated lineages experience a constant in- or efflux of genes that, in turn, continuously trigger the emergence of local clones or novel clonal clusters [24]. Apparently, antimicrobial resistance, and also vancomycin resistance, is disconnected from the clonal success, as shown by Raven et al., disclosing numerous genetic clusters that contained highly related *vanA*-positive and -negative *E. faecium* [25].

Recent publications from Germany describe outbreaks with ST117-type strains and sub-lineages thereof [26,27]. The herein described outbreak revealed a dominant clone of ST80/CT1013, with a *vanB*-type vancomycin resistance operon. It must be noted that all isolates sequenced in this study were selected upon criteria as defined above. Hence, only an incomplete picture of the clonal situation at a given time point is provided. Further, an intra-person VRE variability can influence the interpretation of the results. In our study, we analyzed rectal swabs and clinical specimens of 11 patients with 3 patients showing different ST and CT types between the swabs and the clinical specimens. One of these isolate pairs (with 52 allele differences) was collected on the same day and the isolate pair displaying as many as 379 core genome allele differences was isolated only four months apart. This clearly emphasizes that interpretation of the overall population structure is challenging and it further fuels the discussion on the reliability of a single site and/or single colony picking for outbreak investigations.

Our analyses demonstrated the oligo- or polyclonal nature of VRE outbreaks and that nosocomial transmission occurs as well as constant influx into highly vulnerable settings, which have been described previously [25,28,29,30,31]. Our data on VRE strain variants imported into the hospitals included isolates that are closely related to nation-wide endemic clones (e.g., ST117/CT469) [32]. How this frequent exchange of VRE of certain lineages between geographically distant regions can be explained still remains obscure and is a subject of ongoing investigations.

The abundance of enterococci and the ongoing introduction of VRE or de novo generation are challenging hospitals of all levels of care. As a limitation of our study, we had not isolated VSE; hence, we cannot rule out de novo generation of VRE sourcing the *vanB* gene cluster from commensal and anaerobic bowel bacteria, which has been hypothesized before [33].

In accordance with our analysis, a comprehensive study that was carried out at a university medical center in Southern Germany, and in a region close to the hospitals we investigated, demonstrated the expansion of two sequence types, ST117 and ST80, both *vanB*-type strains [31]. Although Germany lacks a comprehensive molecular surveillance of VRE, the NRC at RKI has carried out ST typing for invasive VRE isolates since 2011 (and cgMLST typing since 2015) and trends can be inferred from these data. Accordingly, increasing numbers of ST117 and ST80 clones were recognized over the years with ST80 starting to expand in 2015 [11]. However, the lineage most dominating the described outbreak, ST80/CT1013, was not recognized as an epidemic clone back then or ever since at the NRC. By applying integrated epidemiological and genomic surveillance, we could demonstrate (i) that this clone was not detected in any other hospital at the time or thereafter and (ii) that ST80/CT1013 was most prominent amongst those VRE that were acquired in the hospital compared to those with influx information, thereby rejecting the hypothesis of constant influx being the major driver of this outbreak as proclaimed by some stakeholders. This valuable information resulted in adjusted IPC measures that were needed to control the outbreak eventually.

Over the course of this outbreak, a plethora of IPC measures were applied and constantly monitored. As evident from publications and the KRINKO recommendation, there is no single measure that will control an outbreak with enterococci. It rather requires a significant effort to (i) comprehensively assess the outbreak situation by setting up a sustained screening strategy, which could be challenging on its own [13,14]; (ii) choose from various options such as case isolation, formation of a VRE outbreak response team, environmental decontamination, antibiotic stewardship, or training and education of staff and visitors, depending on available resources [9,13,29]. Maintaining a high level of preventive measures is not only relevant to contain VRE but was also demonstrated to reduce the spread of other multidrug-resistant bacteria [34]. It might need quite a long time to implement and constantly evaluate the IPC strategy, sometimes several months to even years (this study), for final containment of VRE [29]. To start with, timeliness of reporting, which was 3 days in our case, is one way to rapidly adjust downstream measures. It must be noted that this timespan includes transportation of collected specimens, hence highlighting the advantage of a clinic to own a laboratory unit. Further, our study demonstrates how often screening policies needed adjustment during the course of an outbreak in order to limit drop-outs of VRE detection. In addition, the continuously high positivity rate emphasizes the importance of consistent compliance to eventually bringing VRE rates down to whatever is supposed an “acceptable” level of VRE colonization.

Although the outbreak comprised 2905 cases, screening was not exhaustive throughout the outbreak (Figure S2); thus, the total number of VRE burden (presumably with respect to VRE colonization) is most likely underestimated. Of the patients with no previous contact with either hospital, 8.3% of cases had VRE isolates from sterile body fluids or sites unlikely for *Enterococcus* colonization. Although this does not correspond to hospital-acquired infections (HAI) per definition, the number seems relatively high compared to the results of a recent systematic review and meta-analysis showing a hospital-wide prevalence pooled estimate of 4.6 (95% confidence interval (CI): 2.9–6.7) cases of *Enterococcus* spp. HAI per 1000 hospital patients [35]. The study also emphasized that the pooled estimate of all-cause mortality among patients with hospital-acquired bloodstream infections attributable to VRE was as high as 33.5% (95%CI: 13.0–57.3). This is of particular importance as colonization substantially increases the risk for subsequent infection [36,37]. Hence, this could result in a tremendous individual and economic burden [38], if timely detection of VRE and subsequent interventions in clinical settings are considered less relevant.

## 5. Conclusions

This outbreak investigation showed that a VRE outbreak can reach a tremendous size but can be contained. The integrated epidemiological and genomic assessment of VRE populations in hospital settings is indispensable understanding the nature and dynamics of such complex outbreak situations. These findings will support adjusting infection prevention and control measures, which, applied as a multifaceted bundle, are necessary to contain VRE and limit the spread within and beyond healthcare facilities. That may include implementing measures for the entire hospital, if specialized units are interconnected; performing measure bundles with an adherence of nearly 100%, because nonadherent coworkers prohibit success or subjecting the supervision of adherence to control or self-controlling applications.

## Data Availability

Sequencing data are available from the Sequence Read Archive under accession number: PRJNA851271. Epidemiological data are only available in aggregated, anonymized format and upon special request.

## Supplementary Materials

The following supporting information can be downloaded at: https://www.mdpi.com/article/10.3390/microorganisms10081603/s1, Table S1: Infection prevention and control measures implemented in addition to the regular hygiene plan during a nosocomial outbreak with VRE in two interconnected hospitals in Southern Germany, October 2015–November 2019. Table S2: Core genome MLST-based distance matrix of 134 isolates with epidemiological information about influx or nosocomial transmission from a nosocomial outbreak with VRE in two interconnected hospitals in Southern Germany, October 2015–November 2019. Figure S1: Distribution of clinical specimens among all isolates sequenced (*n* = 397) during a nosocomial outbreak with VRE in two interconnected hospitals in Southern Germany, October 2015–November 2019; Figure S2: VRE screening adherence during a nosocomial outbreak with VRE in two interconnected hospitals in Southern Germany, October 2015–November 2019; Figure S3: VRE positivity rates obtained from screening patients upon admission during a nosocomial outbreak with VRE in two interconnected hospitals in Southern Germany, October 2015–November 2019.
