# Supplementary material for: Controlling an Unprecedented Outbreak with Vancomycin-Resistant Enterococcus faecium in Germany, October 2015 to November 2019
Source: Microorganisms. 2022 Aug 9;10(8):1603. doi: 10.3390/microorganisms10081603 (PMC9412439; doi:10.3390/microorganisms10081603)
Supplement: Supplementary file 1 [file microorganisms-10-01603-s001.zip › Microorganism_BenderJ_VRE_SupplementaryMaterial_revised.pdf]

Supplementary Material

Table S1. Infection prevention and control measures implemented in addition to the regular hygiene plan during a nosocomial outbreak with VRE in two interconnected hospitals in Southern Germany, October 2015 – November 2019.

Infection prevention and control (IPC) measure	
**IPC1: Information about VRE**	Emphasis was first laid on training of nursing staff and doctors, covering topics such as the occurrence of VRE, way of transmission and behaviour in isolation rooms, protective equipment and hand hygiene. VRE information leaflets for patients, dependents, and nursing homes were drafted and distributed. Training was repeated and several other medical supporting groups were included, i.e. physiotherapists, cleaning personnel, personnel from function units, supply personnel, counselling personnel and volunteers. It was aimed and protocolled to train over 90% of the entire personnel.
**IPC2: Disinfection**	Final disinfection of rooms from isolated patients was performed routinely in both hospitals. In 2016, the general cleaning was switched to disinfectant cleaning. For surface disinfection, disinfection-wipes based on oxidizing disinfectant (Incidin TM Oxywipe, Ecolab, Siegsdorf, Germany) were introduced. The A0-value of all pot dishers were raised from 60s to 600s. Disinfection plans were revised in detail and disinfection was trained to staff. Detailed information was given to staff to indicate hand contact surfaces. Decentralized disinfection of nursing equipment, for example wheel chairs, toilet seat raisers, were consequently processed in the central disinfection unit. Equipment that could not reliably be disinfected, like pill organizers handed to patients, were replaced by single-use equipment. The measures were accompanied by multiple and continuous environmental investigations.
**IPC3: Cleaning**	Cleaning worker were supervised by an external hygiene specialist and staff was trained in standard cleaning procedures. To overcome language difficulties pictograms were implemented. At least all supply carts were equipped identically and the entire staff performed cleaning according to the hospital standards. Cleaning was also intensified, as for example, a second cleaning of patient rooms was performed in the afternoon, with emphasis on hand contact surfaces. In spring 2018, an

	extra cleaning of the entire house was carried out. The cleaning personnel was supported by new employees. Last but not least, a self-control program was implemented, where, based on a list with predetermined points, several contact swab cultures were taken by the lead worker. All results of these cultures were reported to the cleaning personnel by hygiene specialists.
**IPC4: Hand hygiene**	In-house training about hand hygiene was performed regularly. During the outbreak, this was intensified and often recapitulated. Further, the hospitals participated in a nationwide study of the German National Reference Center for Surveillance of Nosocomial Infections to monitor the consumption of hand disinfecting agent (study: Hand KISS). Results were reported back regularly. As a control measure, contact swab cultures were taken from the hands of several healthcare workers. These were taken immediately after disinfection, but also on the way to patients. All results were reported back in anonymous manner to the healthcare workers. In October 2017, a sensor-mediated hand disinfection control (Hyhelp) was implemented, which allowed to monitor hand disinfection directly on the wards and give immediate feedback directly to the healthcare workers. Since 2018, the hospitals took part on the nationwide campaign "Aktion saubere Hände" (“Clean Hands Campaign”).
**IPC5: Reconstruction**	A refurbishment of all defective furniture was undertaken between 2017 to 2019, to provide solid surfaces for surface disinfection. The central emergency ward was reconstructed to facilitate surface disinfection and to provide more space. The endoscopy facility was reconstructed. Storage keeping in all hospital wards was revised and closed shelves were installed in 2018. Also, the reconditioning of clinic beds was revised in 2018 resulting in a change from decentral reconditioning to central reconditioning.
**IPC6: Patient isolation**	Patients were isolated in single rooms irrespective of colonization or infection. Restricted access was granted when medically justifiable. Protective equipment such as head covers, masks, disposable gowns, gloves and aprons for staff and visitors was used when entering the room. The patient remained in the room. Disposable items were used whenever possible, otherwise patient-related items were

	disinfected after usage and a final disinfection of the entire room was carried out at patient discharge. Cohort of positive patients was only carried out later in time and then only intermittently.

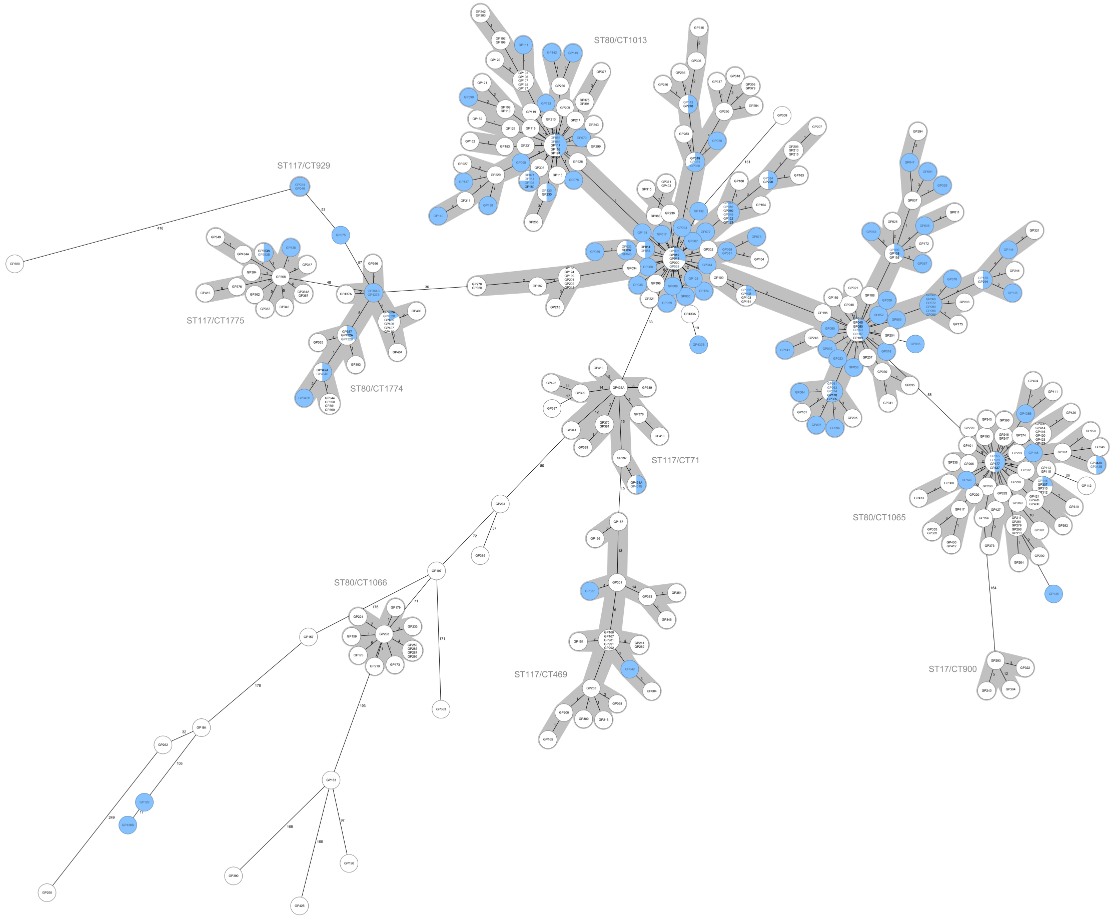

Figure S1. Distribution of clinical specimen among all isolates sequenced (n=397) during a nosocomial outbreak with VRE in two interconnected hospitals in Southern Germany, October 2015 – November 2019. Clinical specimen is depicted in blue, isolates from colonization in white. Sequence types (ST) and complex types (CT) are shown adjacent to the clusters, highlighted in grey, and which represent closely related (difference ≤ 15 alleles) isolates. Numbers adjacent to connecting lines represent alleles difference between isolates.

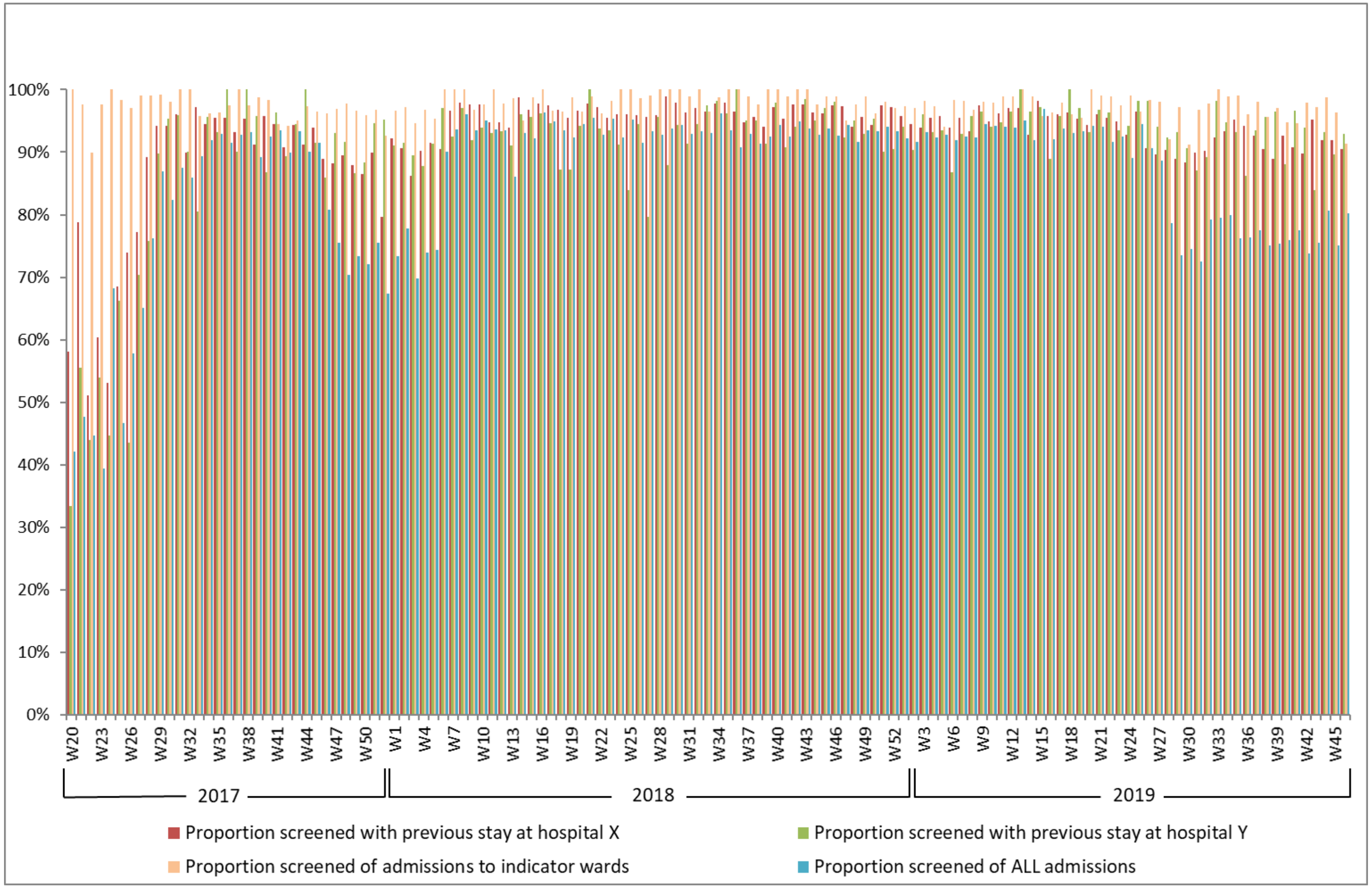

Figure S2. VRE screening adherence during a nosocomial outbreak with VRE in two interconnected hospitals in Southern Germany, October 2015 – November 2019. The percentage of patients screened on indicator wards (salmon), previous admission to hospital X or Y (red and green) and of screened individuals among all patients admitted to either hospital is displayed for the period of May 2017 until November 2019.

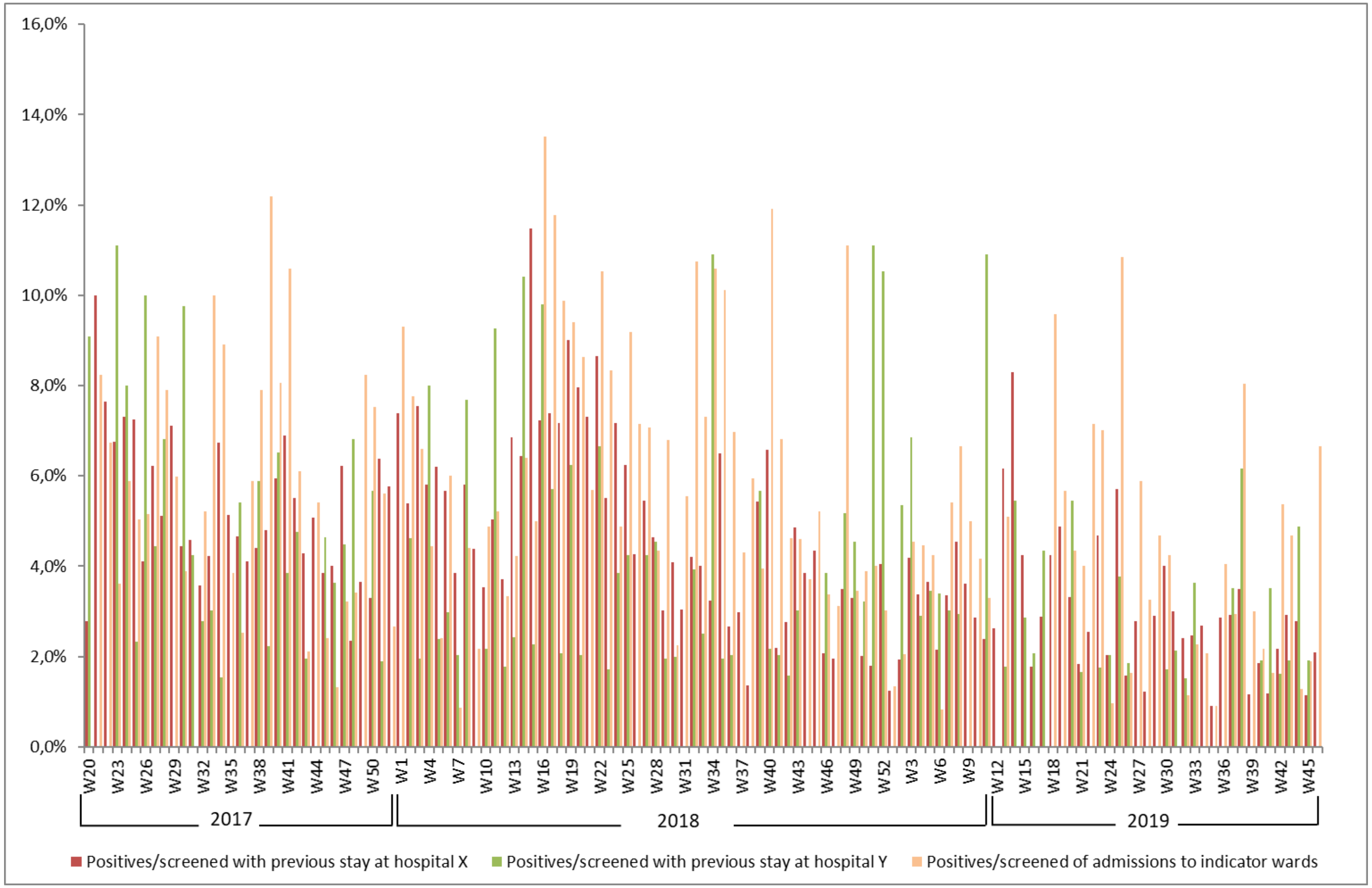

Figure S3. VRE positivity rates obtained from screening patients upon admission during a nosocomial outbreak with VRE in two interconnected hospitals in Southern Germany, October 2015 – November 2019. The percentage of patients screened on indicator wards (~~green~~), previous admission to hospital X or Y (red and ~~grey~~) are displayed for the period of May 2017 until November 2019.
